# Supplementary material for: Dietary Tea Polyphenols Improve Growth Performance and Intestinal Microbiota Under Chronic Crowding Stress in Hybrid Crucian Carp
Source: Animals (Basel). 2025 Jul 5;15(13):1983. doi: 10.3390/ani15131983 (PMC12248730; doi:10.3390/ani15131983)
Supplement: Supplementary file 1 [file animals-15-01983-s001.zip › animals-3678537-supplementary.pdf]

Dietary tea polyphenols improve growth performance and intestinal microbiota under chronic crowding stress in hybrid crucian carp

Zhe Yang[1,2,#], Gege Sun[1,2,#], Jinsheng Tao[1,2], Weirong Tang[1,2], Wenpei Li[1,2], Zehong Wei[1,2], Qifang Yu[1,2,*]

[1] State Key Laboratory of Developmental Biology of Freshwater Fish, Hunan Normal University, Changsha 410081, Hunan, PR China

[2] Yuelushan Laboratory, Changsha, 410128, Hunan, PR China

[#] Contributed equally to this work

[*] Correspondence should be addressed to Qifang Yu (yuqf@hunnu.edu.cn)

1. Microbiomics method

1.1. DNA extraction and PCR amplification

Total microbial genomic DNA was extracted from intestinal contents of the hybrid crucian carp HCC2 samples using the E.Z.N.A.® soil DNA Kit (Omega Bio-tek, Norcross, GA, U.S.) according to manufacturer's instructions. The quality and concentration of DNA were determined by 1.0% agarose gel electrophoresis and a NanoDrop2000 spectrophotometer (Thermo Scientific, United States) and kept at -80 °C prior to further use. The hypervariable region V3-V4 of the bacterial 16S rRNA gene were amplified with primer pairs 338F (5'-ACTCCTACGGGAGGCAGCAG-3') and 806R(5'-GGACTACHVGGGTWTCTAAT-3') by T100 Thermal Cycler PCR thermocycler (BIO-RAD, USA). The PCR reaction mixture including 4 μL 5 × Fast Pfu buffer, 2 μL 2.5 mM dNTPs, 0.8 μL each primer (5 μM), 0.4 μL Fast Pfu polymerase, 10 ng of template DNA, and ddH_2O to a final volume of 20 μL. PCR amplification cycling conditions were as follows: initial denaturation at 95 °C for 3 min, followed by 27 cycles of denaturing at 95 °C for 30 s, annealing at 55 °C for 30 s and extension at 72 °Cfor 45 s, and single extension at 72 °C for 10 min, and end at 4 °C. The PCR product was extracted from 2% agarose gel and purified using the PCR Clean-Up Kit (YuHua, Shanghai, China) according to manufacturer's instructions and quantified using Qubit 4.0 (Thermo Fisher Scientific, USA) .

1.2. Illumina sequencing

Purified amplicons were pooled in equimolar amounts and paired-end sequenced on an Illumina Nextseq2000 platform (Illumina, San Diego,USA) according to the standard protocols by Majorbio Bio-Pharm Technology Co. Ltd. (Shanghai, China).

1.3. Data processing

Raw FASTQ files were de-multiplexed using an in-house perl script, and then quality-filtered by fastp version 0.19.6 and merged by FLASH version 1.2.7 with the following criteria:

(i) The reads were truncated at any site receiving an average quality score of <20 over a 50 bp sliding window, and the truncated reads shorter than 50 bp were discarded, reads containing ambiguous characters were also discarded;

(ii) Only overlapping sequences longer than 10 bp were assembled according to their overlapped sequence. The maximum mismatch ratio of overlap region is 0.2. Reads that could not be assembled were discarded;

(iii) Samples were distinguished according to the barcode and primers, and the sequence direction was adjusted, exact barcode matching, 2 nucleotide mismatch in primer matching. Then the optimized sequences were clustered into operational taxonomic units (OTUs) using UPARSE 11 with 97% sequence similarity level. The most abundant sequence for each OTU was selected as a representative sequence. The OTU table was manually filtered, i.e., chloroplast sequences in all samples were removed. To minimize the effect of sequencing depth on the measurement of alpha and beta diversity, the number of 16S rRNA gene sequences in each sample was flattened by the minimum number of sample sequences, which still yielded an average Good's coverage of 98.35%. Taxonomic annotation of OTU species was performed by using RDP classifier (http://rdp.cme.msu.edu/, version 2.11) compared with Silva 16S rRNA gene database (v138). The confidence threshold was 70%. The community composition of each sample was calculated at different species classification levels.

1.4. Statistical Analysis

Bioinformatic analysis of the gut microbiota was carried out using the Majorbio Cloud platform (https://cloud.majorbio.com). Based on the OTUs information, rarefaction curves and alpha diversity indices including observed OTUs, Chao1 richness, Shannon index, observed richness and Good’s coverage were calculated with Mothur v1.30.1. The similarity among the microbial communities in different samples was determined by principal coordinate analysis (PCoA) based on Bray-curtis dissimilarity using Vegan v2.5-3 package. The PERMANOVA test was used to assess the percentage of variation explained by the treatment along with its statistical significance using Vegan v2.5-3 package. The linear discriminant analysis (LDA) effect size (LEfSe) (http://huttenhower.sph.harvard.edu/LEfSe) was performed to identify the significantly abundant taxa (phylum to genera) of bacteria among the different groups (LDA score > 3.0).

2. Supplementary figures

2.1 Figure S1 Pan and Core curves

Pan/Core species analysis is designed to characterize the trends in the total species count and core species abundance as sample size expands. The approach is commonly employed to evaluate both environmental species richness and core species representation, enabling a determination of whether the sequencing sample size is adequate for achieving the study's objectives.

Fig S1. The Pan (A) and Core (B) curves of the hybrid crucian carp intestinal

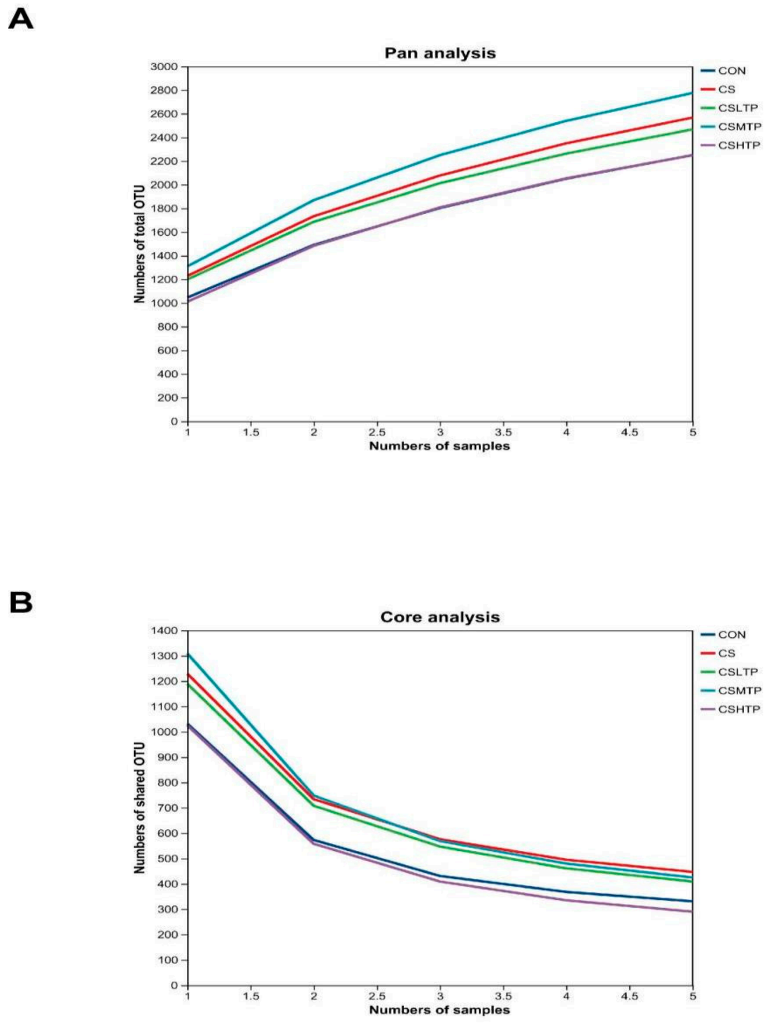

microbiota in response to crowding stress at the OTU level.

The x-axis denotes the number of observed samples, while the y-axis indicates the number of shared or core species within all samples under a specific grouping category.

2.2 Figure S2 rarefaction curve

The dilution curve is drawn by randomly sampling a certain number of sequences from the sample and calculating the Alpha diversity index (such as Sobs index) corresponding to these sequences in the sample. The amount of sampled data is taken as the abscissa and the value of the Alpha diversity index as the ordinate. Whether the curve has reached a plateau is used to determine whether the sequencing data volume of this test is sufficient.

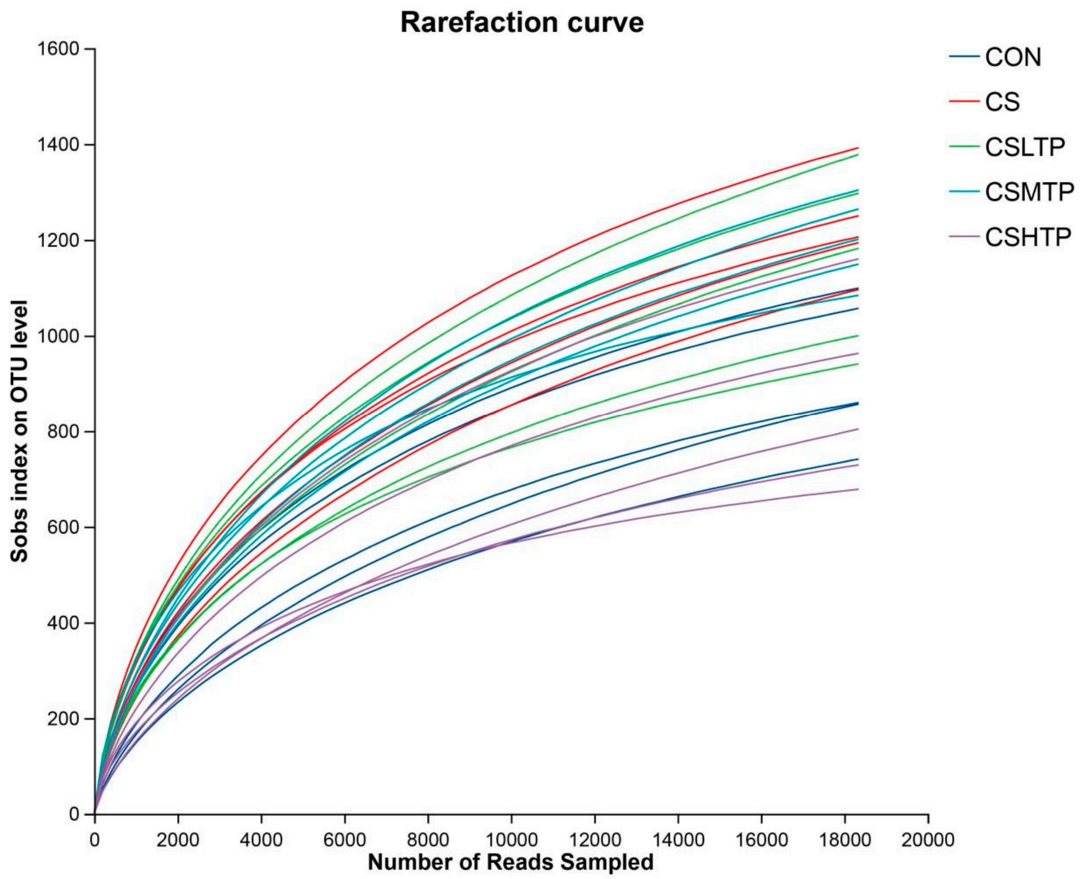

Fig S2. The rarefaction curve of the hybrid crucian carp intestinal microbiota in response to crowding stress at the OTU level.

The x-axis denotes the quantity of randomly selected sequencing data, while the y-axis indicates the number of observed species (Sobs index) on OTU level.

3. Supplementary tables

3.1. Table S1 The certification of tea polyphenols

Tea polyphenols (TPs) were provided by the National Research Center of Engineering Technology for Utilization of Functional Ingredients from Botanicals, Hunan Agricultural University, Changsha, China. The certification for TPs is shown in Table S1.

Table S1 The certification of tea polyphenols.

Examination Items	Unit	Specification	Result
Appearance		Brown powder	Confirmed
Total Tea Polyphenols	%	＞= 98.0%	98.50%
Epigallocatechin-3-gallate (EGCG)	%	＞= 50.0%	52.20%
Epicatechin (EC)	%		11.9%
Epicatechin gallate (ECG)	%		9.8%
Epigallocatechin (EGC)	%		8.4%
Gallocatechin gallate (GCG)	%		5.0%
Catechin (C)	%		4.7%
Gallocatechin (GC)	%		3.4%
Gallic acid (GA)	%		3.1%
Caffeine	%	＜=2.0%	0.80%
Water Content	%	＜=5.0%	3.20%
Ash	%	＜=1.0%	0.50%
Granularity		＞= 95.0% through 80 Mesh	Confirmed
Lead	PPM	＜=5.0	Confirmed
As	PPM	＜=2.0	Confirmed
Total Bacterial	cfu/g	＜=1000	Confirmed
Mold & Yeast	cfu/g	＜=100	Confirmed
Colibacillus		Negative	Negative
Salmonella		Negative	Negative

3.2. Table S2 The Venn figure data

The first column represents the shared or unique group information (Group_lable). For instance, in the above table, "CON only" indicates the species that are unique to the CON group category, while "CON&CS" represents the species shared by both the CON and CS groups. The second column shows the number of species (Species_num), indicating the number of shared or unique species.

Table S2 The Venn figure data at OTUs level

Group_lable	Species_num
CSMTP only	631
CSHTP & CSLTP & CSMTP	31
CS & CSHTP & CSLTP & CSMTP	134
CON & CSHTP & CSLTP & CSMTP	45
CS & CSHTP & CSMTP	42
CS & CSLTP	107
CSLTP & CSMTP	81
CON & CSHTP & CSLTP	25
CON & CS & CSLTP	80
CON only	349
CON & CSHTP & CSMTP	31
CON & CS & CSHTP & CSLTP	81
CON & CS & CSMTP	67
CON & CS & CSHTP	32
CON & CS & CSHTP & CSMTP	47
CON & CSLTP	50
CON & CSLTP & CSMTP	30
CSHTP only	250
CON & CSHTP	30

CS only	415
CON & CS & CSLTP & CSMTP	137
CON & CS	72
CSLTP only	359
CSHTP & CSLTP	51
CS & CSMTP	83
CON & CS & CSHTP & CSLTP & CSMTP	1061
CS & CSHTP & CSLTP	56
CSHTP & CSMTP	81
CS & CSLTP & CSMTP	107
CON & CSMTP	45
CS & CSHTP	55

3.3. Table S3 and S4 Statistical table of difference test

The significance test of inter-group differences is mainly used to analyze whether there are differences in microbial composition between the control group and the treatment group, and to identify the microorganisms with significant differences. These different microorganisms may be key species responding to environmental changes, and thus can be used to find microbial biomarkers for the control group and the treatment group, helping to build predictive models.

Table S3 Statistical table of difference test at phylum level

Species name	CON-Mea	CON-Sd(%	CS-Mean(%	CS-Sd(%)	CSHTP-Me
p__Proteobacteria	29.03	9.932	37.16	10.26	41.17
p__Firmicutes	36.61	22.68	12.9	4.757	21.26
p__Actinobacteriota	10.15	7.085	17.08	1.586	12.15
p__Chloroflexi	8.583	3.982	13.45	4.709	6.784
p__Cyanobacteria	1.068	0.6907	1.554	0.7272	10.6
p__Desulfobacterota	2.121	1.253	3.901	1.413	1.275
p__Myxococcota	0.5102	0.2495	3.829	1.752	0.6956
p__Planctomycetota	1.603	1.24	1.646	0.5503	1.658
p__Fusobacteriota	5.452	11.4	0.5637	0.5853	0.3947
p__Verrucomicrobiota	1.024	0.5767	1.491	0.5541	0.7719
p__Patescibacteria	0.3565	0.1523	1.807	0.3587	0.8875
p__Bacteroidota	0.7937	1.194	0.05669	0.04472	0.1123
p__Bdellovibrionota	0.8199	0.6897	1.003	0.2562	0.8679
p__Acidobacteriota	0.6291	0.2048	1.578	1.277	0.3772
p__Dependentiae	0.4328	0.3034	0.8133	0.2129	0.4688

(Please double-click on the above picture to view the detailed content.)

Note: Mean (%) represents the average relative abundance of the species in different groups; Sd (%) represents the standard deviation. The P_value represents the probability of a false positive result obtained from the Kruskal-Wallis H test, where a value of $P < 0.05$ indicates a significant difference. The P_adjust represents the adjusted P value after correction.

Table S4 Statistical table of difference test at genus level

Species na	CON-Mea	CON-Sd(%	CS-Mean(%	CS-Sd(%)	CSHTP-Me
g__norank_	9.795	6.64	15.89	10.03	7.952
g__Mycoba	4.225	3.8	6.229	0.8534	6.005
g__Lactocc	21.48	30.03	1.106	1.441	1.696
g__Methylc	3.89	2.818	5.3	1.426	4.744
g__norank_	3.307	2.794	3.73	1.092	3.918
g__norank_	2.703	2.099	4.529	1.813	2.155
g__Pseudo	0.09922	0.1146	0.1505	0.1722	7.401
g__norank_	2.392	1.042	4.232	2.824	0.8112
g__Clostrid	1.582	1.001	2.041	0.9681	2.115
g__Pantoea	4.341	9.708	0	0	4.864
g__Desulfo	1.337	1.114	2.743	1.109	0.833
g__norank_	1.634	1.533	2.933	1.487	0.5942
g__Bacillus	0.5887	0.3211	0.676	0.3913	6.056
g__Rombo	0.5909	0.5549	2.085	2.636	2.197
g__Cetoba	5.379	11.44	0.5321	0.5959	0.3892
g__norank_	1.173	0.77	1.34	0.4658	1.494
g__Methylc	1.365	2.339	0.6084	0.3711	0.9802
g__norank_	0.04034	0.02742	2.375	1.614	0.1723
g__norank_	1.014	0.2016	1.776	0.79	0.5691
g__Paenicl	1.671	3.235	0.6062	0.6683	1.477
g__Alsobac	0.4884	0.4707	0.7032	0.4137	0.9605
g__Chrooc	0.02944	0.05079	0.06106	0.1	4.411
g__norank_	0.1439	0.103	0.2878	0.1403	1.215
g__Sphingo	0.2573	0.2202	0.676	0.7648	1.159
g__Hyphor	0.5746	0.2904	0.8744	0.3758	0.5004
g__alphaI_	0.2682	0.1647	0.9562	0.226	0.5473
g__Fictibac	0.04579	0.03923	0.1014	0.06223	2.831
g__norank_	0.4634	0.2498	0.9027	0.3438	0.3696
g__Bacterc	0.2257	0.4683	0.0229	0.02228	0.008722
g__Auranti	0.06542	0.03987	0.4481	0.4173	0.5898
g__Pedom	0.5615	0.4946	0.64	0.1996	0.3903
g__norank_	0.1297	0.03785	0.7643	0.1316	0.35
g__Chrooc	0	0	0.004361	0.009752	2.429
g__norank_	0.1625	0.09846	0.6825	0.2305	0.3554
g__norank_	0.2061	0.1254	0.5124	0.2627	0.2933
g__Vogese	0.2159	0.2204	0.3761	0.4516	0.6749
g__Exiguob	1.33	2.674	0.1973	0.1444	0.2682
g__Leptoly	0.008722	0.0113	0.07741	0.09398	1.947
g__norank_	0.5659	0.8135	0.8842	1.189	0.03489

(Please double-click on the above picture to view the detailed content.)

Note: Mean (%) represents the average relative abundance of the species in different groups; Sd (%) represents the standard deviation. The P_value represents the probability of a false positive result obtained from the Kruskal-Wallis H test, where a value of $P < 0.05$ indicates a significant difference. The P_adjust represents the adjusted P value after correction.
